# Integration of Convergent Sensorimotor Inputs Within Spinal Reflex Circuits in Healthy Adults

**DOI:** 10.3389/fnhum.2020.592013

**Published:** 2020-11-26

**Authors:** Alejandro J. Lopez, Jiang Xu, Maruf M. Hoque, Carly McMullen, Trisha M. Kesar, Michael R. Borich

**Affiliations:** ^1^Neural Plasticity Research Laboratory, Division of Physical Therapy, Department of Rehabilitation Medicine, Emory University, Atlanta, GA, United States; ^2^Motion Analysis Laboratory, Division of Physical Therapy, Department of Rehabilitation Medicine, Emory University, Atlanta, GA, United States; ^3^Department of Rehabilitation Medicine, Tongji Hospital, Tongji Medical College, Huazhong University of Science and Technology, Wuhan, China

**Keywords:** spinal neurophysiology, H-reflex, transcranial magnetic stimulation, lower motor neuron, short interval facilitation, long interval facilitation

## Abstract

The output from motor neuron pools is influenced by the integration of synaptic inputs originating from descending corticomotor and spinal reflex pathways. In this study, using paired non-invasive brain and peripheral nerve stimulation, we investigated how descending corticomotor pathways influence the physiologic recruitment order of the soleus Hoffmann (H-) reflex. Eleven neurologically unimpaired adults (9 females; mean age 25 ± 3 years) completed an assessment of transcranial magnetic stimulation (TMS)-conditioning of the soleus H-reflex over a range of peripheral nerve stimulation (PNS) intensities. Unconditioned H-reflex recruitment curves were obtained by delivering PNS pulses to the posterior tibial nerve. Subsequently, TMS-conditioned H-reflex recruitment curves were obtained by pairing PNS with subthreshold TMS at short (−1.5 ms) and long (+10 ms) intervals. We evaluated unconditioned and TMS-conditioned H-reflex amplitudes along the ascending limb, peak, and descending limb of the H-reflex recruitment curve. Our results revealed that, for long-interval facilitation, TMS-conditioned H-reflex amplitudes were significantly larger than unconditioned H-reflex amplitudes along the ascending limb and peak of the H-reflex recruitment curve. Additionally, significantly lower PNS intensities were needed to elicit peak H-reflex amplitude (Hmax) for long-interval facilitation compared to unconditioned. These findings suggest that the influence of descending corticomotor pathways, particularly those mediating long-interval facilitation, contribute to changing the recruitment gain of the motor neuron pool, and can inform future methodological protocols for TMS-conditioning of H-reflexes. By characterizing and inducing short-term plasticity in circuitry mediating short- and long-interval TMS-conditioning of H-reflex amplitudes, future studies can investigate supraspinal and spinal circuit contributions to abnormal motor control, as well as develop novel therapeutic targets for neuromodulation.

## Introduction

Coordination between descending corticomotor and peripheral sensory inputs that converge at the spinal segmental level is needed for typical motor control. The monosynaptic spinal reflex pathway, which mediates stretch reflexes, consists of group Ia afferent fibers that make excitatory connections with alpha (α)-motoneurons at the spinal segmental level (Meunier and Pierrot-Deseilligny, 1989; Pierrot-Deseilligny and Mazevet, 2000; Chen et al., 2003; Wang et al., 2012). The Hoffman (H-) reflex is the electrical analog of the monosynaptic stretch reflex that bypasses the muscle spindle and fusimotor activity, and is used to evaluate the excitability of the monosynaptic spinal reflex pathway (Gassel, 1969; Schieppati, 1987; Knikou, 2008). Unlike the spinal reflex pathway, volitional movement is generated when pyramidal neurons in the primary motor cortex (M1) activate and conduct descending excitatory signals to spinal lower motor neurons (LMNs) that innervate target muscle groups. Subcortical and brainstem-mediated descending pathways can also project onto spinal LMNs, thus influencing the excitability of the spinal LMN pool (Ross et al., 1975, 1976; Bagust et al., 1985; Nielsen and Petersen, 1995b; Buschges, 2017). Therefore, spinal LMNs are the final common pathway for all motor output, whether reflexive or volitional. Neuropathologies such as stroke or multiple sclerosis can compromise motor control circuitry resulting in altered spinal reflex activity and impaired motor control (Bakheit et al., 2005; Schindler-Ivens et al., 2008; Perez and Cohen, 2009; Bhagchandani and Schindler-Ivens, 2012). To better understand neural mechanisms of abnormal motor control, descending corticomotor influences on spinal α-motoneurons in the unimpaired nervous system need to be understood.

Reflexive motor output that occurs when a mixed peripheral nerve is electrically stimulated (Walsh et al., 1998; Rossini et al., 2015) is commonly measured in upper and lower extremity muscles using surface electromyography (EMG) (Granit and Job, 1952; Gassel and Diamantopoulos, 1966; Knikou, 2008; Burke, 2016). H-reflexes are evoked by low-intensity surface electrical stimulation of a mixed peripheral nerve (peripheral nerve stimulation, PNS), which trans-synaptically excites the LMN pool, and are influenced by pre- and post-synaptic mechanisms (Komiyama et al., 1999; Milanov, 2000). H-reflex responses recorded over a range of PNS intensities generate the H-reflex recruitment curve, which provides useful information regarding reflex gain and metrics of spinal reflex excitability such as the peak H-reflex amplitude (Funase et al., 1994; Mazzocchio et al., 2001). Understanding the shape (e.g., slope) and behavior (e.g., leftward shift) of the H-reflex recruitment curve may provide novel insights into the physiologic processes that modulate the recruitment order and gain of LMN pools. However, a current limitation of classic H-reflex methodology is the inability to differentially assess the influence of cortical, subcortical, brainstem, and intra-spinal inputs that target the LMN pool. The influence of Renshaw cells (Ross et al., 1972, 1976) and presynaptic inhibition (Meunier and Pierrot-Deseilligny, 1998; Milanov, 2000) on the monosynaptic spinal reflex pathway has been previously studied using paired PNS methods or antagonist muscle activation. However, methodologies to parse out contributions of different *descending* corticomotor inputs (e.g., direct corticospinal tract projections from the motor cortex versus polysynaptic descending projections that travel through brain stem centers) on LMN pools are lacking. Additionally, methodologies that investigate Renshaw cells or presynaptic inhibition do not evaluate the influence of descending corticomotor projections. Hence, alternative techniques are needed to determine the influence of descending corticomotor inputs on the recruitment gain of the LMN pool.

Transcranial magnetic stimulation (TMS) delivered over the primary motor cortex (M1) is used to non-invasively characterize excitability of descending corticomotor pathways (Barker et al., 1985; Hallett, 2007; Groppa et al., 2012). A single supra-threshold TMS pulse delivered over M1 elicits a motor evoked potential (MEP) in the contralateral targeted muscle (Barker et al., 1985; Rossini et al., 2015). Although the amplitude of the MEP provides an overall index of corticomotor excitability, standalone TMS is unable to specifically characterize contributions of different descending corticomotor pathways that influence spinal circuit activity.

Combining PNS with subthreshold TMS provides a unique tool to index descending corticomotor influences on spinal reflex excitability (Nielsen et al., 1993; Nielsen and Petersen, 1995b; Geertsen et al., 2011). When a sub-threshold TMS conditioning pulse is delivered before or after PNS, the H-reflex response is typically increased. Short-interval facilitation (SIF) also often referred to as ’short latency facilitation of the H-reflex occurs when a subthreshold TMS pulse is delivered 1–5 ms after a PNS pulse, allowing the direct, fastest descending volley to arrive at the spinal LMN pool prior to the afferent signal, and enhance the H-reflex amplitude by modulating the excitability of the LMN pool (Nielsen and Petersen, 1995b; Gray et al., 2017). Long-interval facilitation (LIF), also referred to as long latency facilitation, occurs when a TMS pulse is delivered before PNS, allowing indirect, slower descending volleys to arrive prior to the afferent signal (Nielsen and Petersen, 1995b; Gray et al., 2017). Thus, SIF and LIF provide measures to non-invasively probe the specific sites and mechanisms of neuromotor circuit connections between cortical and spinal circuitry, which are previously poorly understood.

A single PNS intensity is commonly used when evaluating SIF and LIF (Petersen et al., 2003; Geertsen et al., 2010, 2011). In previous work, PNS intensities were set to elicit an H-reflex amplitude equivalent to 10–30% of the maximal muscle response (Mmax) to investigate both SIF and LIF (Nielsen et al., 1993; Nielsen and Petersen, 1995b; Pyndt and Nielsen, 2003; Geertsen et al., 2010), with the H-reflex amplitude (typically) on the ascending limb of the recruitment curve considered sensitive to facilitatory H-reflex conditioning (Di Lazzaro and Rothwell, 2014; Gray et al., 2017; Taube et al., 2017). Changes in the strength of output from the motor neuron pool can be attributed to different excitation thresholds of afferent fibers and motor axons (Funase et al., 1994; Pierrot-Deseilligny and Mazevet, 2000; Cecen et al., 2018). Additionally, different descending corticomotor pathways that are recruited or become activated due to varying TMS intensity, muscle activation state, or postural activation can influence the activation threshold of different motor neuron pools (Bawa and Lemon, 1993; Costa et al., 2011). While the influence of TMS parameters (e.g., intensity, direction of induced current) on H-reflex facilitation have been explored in previous studies (Niemann et al., 2018), to our knowledge, the effect of PNS intensity on TMS-conditioning of the H-reflex has not been previously reported. We posit that evaluating the effects of TMS-conditioning at multiple PNS intensities across the H-reflex recruitment curves can provide novel insights and a more comprehensive characterization of the mechanisms underlying direct and indirect descending cortical connections that influence spinal segmental reflex circuitry.

Therefore, the overall objective of this study was to investigate the influence of descending corticomotor and peripheral sensory inputs on the physiologic recruitment order of soleus H-reflexes. To characterize different recruitment profiles in response to the integration of sensorimotor inputs onto the motor neuron pool, we evaluated TMS-conditioning of the H-reflex across a range of PNS intensities. We hypothesized that short and long interval facilitation of soleus H-reflexes will occur across multiple PNS intensities, and that the introduction of descending corticomotor inputs will change the characteristics of the H-reflex recruitment curve.

## Materials and Methods

### Participants

Eleven young, neurologically unimpaired participants (9 females; mean age 25 ± 3 years) were recruited and completed the experimental protocol. Exclusion criteria included: (1) any known neurologic, neurodegenerative, orthopedic or musculoskeletal disorder, or psychiatric diagnosis, (2) outside the age range of 18–35 years old, or (3) contraindications to TMS (Rossini et al., 2015). Each participant provided written informed consent in accordance with the Declaration of Helsinki. Study procedures were approved by the Emory University Institutional Review Board (IRB#00067708).

### Experimental Design

Participants completed a single experimental session lasting approximately 3 h, and study procedures were completed at a similar time of day (± 2 h) across participants to account for potential circadian influences. Participants were asked to refrain from strenuous physical activity for 12 h before the session to prevent potential excitability changes induced by strenuous physical activity (Walton et al., 2003; Cerqueira et al., 2006). The right soleus muscle was tested in all participants. Measurements were obtained with the participant seated in a semi-recumbent position with hips and knees in 30° of flexion and the ankles secured in neutral position in a paired rigid boot. Proper participant positioning was ensured using inelastic straps at mid-point of the shank (or lower leg) and thigh bilaterally to prevent hip rotation and abduction.

### Electromyography Procedures

Following standard skin preparation procedures, two surface electrodes (2 cm diameter, EL503 Biopac Systems Inc., Goleta, CA) were affixed to the skin overlying the posterolateral aspect of the right soleus and the tibialis anterior (TA) muscle belly. A ground electrode was placed over the ipsilateral lateral malleolus. To verify proper EMG sensor placement, participants were asked to contract their right soleus and TA while an experimenter confirmed EMG activation (Biopac Systems AcqKnowledge Software Version 4.4). After verifying proper EMG sensor placement, soleus EMG activity was measured during 30 s of quiet standing, and the average EMG amplitude was recorded and used as a target for background EMG during the remaining experimental procedures.

### Unconditioned Soleus H-Reflex Recruitment Curve

Soleus H-reflexes were evoked by stimulating the tibial nerve within the popliteal fossa using a monopolar electrode (round, 2.5 cm), with the anode (square, 5 cm) placed at the midline proximal to the patella (Biopac Systems Inc., Goleta, CA). The stimulating electrodes were self-adhering carbon rubber electrodes. With the participant lying prone, single pulses were delivered at random intervals as the experimenter moved the cathode within the posterolateral popliteal fossa to determine the optimal location to elicit H- and M-waves along with a pure plantar flexion response. Next, with the participant sitting in the semi-reclined test position, threshold stimulating intensities (lowest intensity where an H-reflex response was visible and lowest intensity where the Mmax was observed) were obtained. To control for the effects of varying background EMG activation on spinal and cortical excitability (Nielsen et al., 1993; Guzman-Lopez et al., 2015), participants were requested to maintain the right soleus background EMG activity at a low-level [matched to the level of each individual’s voluntary EMG during quiet standing, ∼10% maximum voluntary contraction (MVC)] by plantarflexing into a small block placed in the rigid boot (Gray et al., 2017). Real-time visual feedback of ongoing soleus root mean square EMG was provided on a computer monitor placed in front of the participant to ensure consistent soleus activity levels during data collection.

Soleus H-reflex and M-wave recruitment curve stimulation intensities were defined by the lowest PNS intensity eliciting an observable (0.1 mV peak-to-peak amplitude) H-reflex response (H-threshold), and the highest PNS intensity resulting in a plateau of the M-wave (Mmax) (Knikou, 2008; Burke, 2016). To collect the H-reflex recruitment curve, approximately 50 stimulation pulses were delivered at increasing intensities (0.2–1 mA) with a variable inter-pulse interval ranging from 4 to 8 s. A 6th order polynomial curve was fit to the individual H-reflex and M-wave responses, generating an H-reflex/M-wave recruitment curve. Using the polynomial curve fit, we extracted H-reflex and M-wave amplitudes equivalent to 20% Mmax, Mmax, H-threshold, 50% Hmax, Hmax, 150% Hmax, and H-curve-endpoint, and used the values from these intensity conditions as the primary outcome measures. The H-threshold was defined as the intensity along the ascending limb of the H-reflex recruitment curve at which consecutive PNS pulses elicited visible H-reflexes. Fifty percent Hmax was defined as the midway point along the ascending limb of the H-reflex recruitment between H-threshold and Hmax. One hundred and fifty percent Hmax was defined as the midway point along the descending limb of the H-reflex recruitment curve between Hmax and H-curve-endpoint. The H-curve-endpoint was defined as the intensity along the descending limb of the H-reflex recruitment curve at which consecutive PNS pulses did not elicit a visible H-reflex.

### TMS Procedures

To identify the M1 hotspot for the right soleus muscle, MEPs were elicited with single TMS pulses delivered with a custom 70 mm figure-of-eight batwing coil (Magstim Company Ltd., Dyfed, United Kingdom) connected to a monophasic stimulator (Magstim 200^2^) (Gray et al., 2017; Kesar et al., 2018). The hotspot was defined as the optimal coil position that elicited the largest MEP response from the right soleus muscle at a fixed suprathreshold stimulation intensity. Consistency and accuracy in coil placement were maintained using stereotaxic neuronavigation using a standard brain template (Brainsight v. 2.2.14, Rogue Research Inc., Canada). Active motor threshold (AMT) was determined as the lowest stimulator intensity needed to evoke a soleus MEP of ≥ 100 μV peak-to-peak amplitude in at least 3 out of 5 trials during a volitional contraction (∼10% MVC) (Gray et al., 2017). AMT intensity values ranged from 41 to 69% MSO (mean = 53% ± 9.3% MSO).

### TMS-Conditioned H-Reflex Recruitment Curves

**FIGURE 1:**
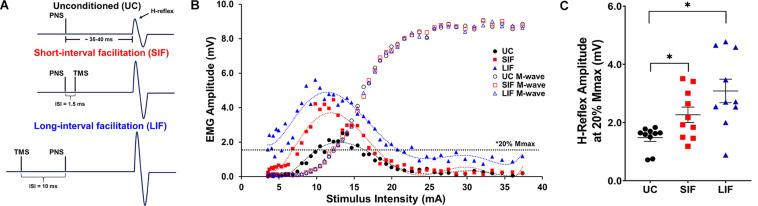
**(A)** Schematic representation of stimulation timing for unconditioned (UC) soleus H-reflex response, and expected short-interval facilitation (SIF) and long-interval facilitation (LIF) of soleus H-reflexes. Inter-stimulus intervals (ISIs) of –1.5 and +10 ms were used to elicit SIF and LIF of soleus H-reflexes, respectively. **(B)** Unconditioned (UC), short-interval facilitation (SIF), and long-interval facilitation (LIF) recruitment curves were plotted from a representative participant. A 6th order polynomial curve was fitted for each recruitment curve and used to extract H-reflex and M-wave amplitudes at multiple intensities across the curve. Note facilitation of the H-reflex for the SIF and LIF recruitment curves compared to the UC recruitment curve, and an apparent leftward shift of the SIF and LIF recruitment curves. **(C)** The graph above shows the mean and standard error of H-reflex amplitudes (mV) at 20% Mmax (*N* = 10) for the UC (1.60 ± 0.15), SIF (2.36 ± 0.29), and LIF (3.29 ± 0.38) conditions. H-reflex amplitudes were significantly increased for both SIF (*p* < 0.03) and LIF (*p* = 0.001) compared to UC.

To investigate TMS-conditioning of the soleus H-reflex, sub-threshold TMS (90% AMT) was delivered at two different inter-stimulus intervals relative to electrical stimulation of the tibial nerve. To elicit SIF, the TMS pulse was delivered 1.5 ms after delivery of PNS (−1.5 ms) and a TMS pulse was delivered 10 ms prior to PNS (+ 10 ms) to index LIF (Figure 1A). Both inter-stimulus intervals have been shown to reliably elicit significant H-reflex facilitation in participants with similar demographic characteristics (Gray et al., 2017). During TMS-conditioning experiments, each recruitment curve dataset was recorded by sequentially delivering interleaved unconditioned (UC) and conditioned (SIF, LIF) pulses (ex: UC_*n*_, SIF_*n*_, LIF_*n*_, UC_*n*__+__1_, SIF_*n*__+__1_, LIF_*n*__+__1_, …) with increasing PNS intensity. The same range of PNS intensities were used for the initial UC H-reflex recruitment curve, as well as the SIF and LIF curves.

### Calculation of H-Reflex and M-Response Amplitude, Hmax, and Mmax

Peak-to-peak amplitude for H-reflex and M-wave responses were identified for each trial, and used to generate the unconditioned and TMS-conditioned recruitment curves (Figure 1B). Evoked responses that failed to exceed background EMG threshold (0.05 mV) were excluded from the analysis. We fit a polynomial curve (6th order) to each individuals’ unconditioned, SIF, and LIF H-reflex recruitment curve dataset (*R*^2^-values between 0.8 and 0.99). Hmax and Mmax were calculated as the average of the three largest H-reflex or M-wave amplitudes, respectively (Gray et al., 2017). TMS-conditioned H-reflex recruitment curves (SIF and LIF) were analyzed using the same methods as unconditioned H-reflexes. All conditioned and unconditioned H-reflex amplitudes were plotted as a function of PNS intensity.

### Determination of Magnitude of TMS-Induced Facilitation

The unconditioned H-reflex recruitment curve was used to find the intensity that elicited an H-reflex amplitude that was equivalent to about 20% of Mmax, as H-reflexes of this size have been shown to be sensitive to inhibitory and facilitatory conditioning (Crone et al., 1990; Nielsen and Petersen, 1995b; Geertsen et al., 2011). TMS-conditioned H-reflex amplitudes for SIF and LIF were also recorded at the same PNS intensity (derived using the UC H-reflex recruitment curve), and used to compute the magnitude of facilitation. Thus, for each participant, the same PNS intensities were delivered for all 3 H-reflex recruitment curves—unconditioned, SIF, and LIF. The magnitude of facilitation for soleus H-reflexes was expressed as the H-reflex amplitude normalized to each individual’s Mmax for UC, SIF, and LIF.

**FIGURE 2:**
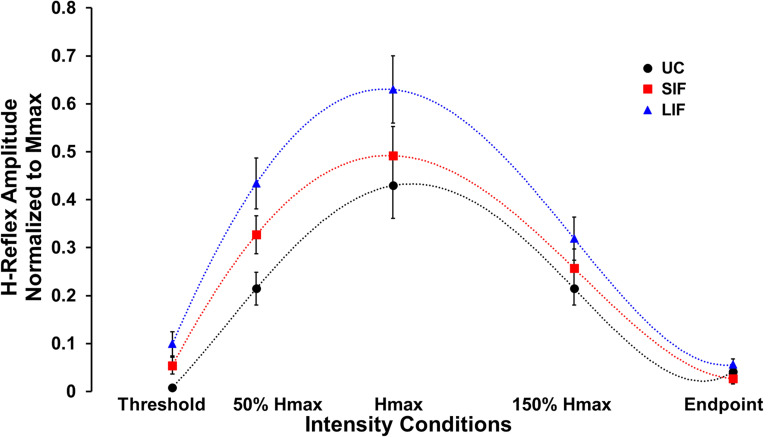
Average H-reflex amplitude normalized to Mmax for each condition (*N* = 10) at five different PNS intensity conditions (Threshold, 50% Hmax, Hmax, 150% Hmax, and Endpoint) along the unconditioned (UC), short-interval facilitation (SIF), and long-interval facilitation (LIF) recruitment curves. A 6th order polynomial curve was fitted for each recruitment curve and used to extract H-reflex and M-wave amplitudes at multiple intensities across the curve.

**FIGURE 3:**
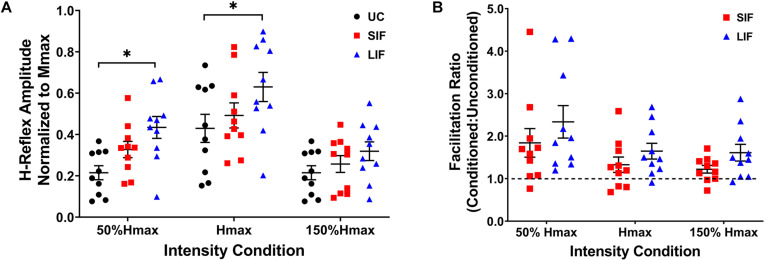
**(A)** The graph shows the mean and standard error for H-reflex amplitudes normalized to Mmax for unconditioned (UC), short-interval facilitation (SIF), and long-interval facilitation (LIF) at three PNS intensity conditions along the ascending (50% Hmax), peak (Hmax), and descending (150% Hmax) portions of the H-reflex recruitment curve. Significant facilitation was observed at 50% Hmax for LIF (0.434 ± 0.053) compared to UC (0.215 ± 0.034), and at Hmax for LIF (0.630 ± 0.070) compared to UC (0.430 ± 0.068). *p < 0.05. **(B)** The graph above shows the mean and standard error for the facilitation ratio, calculated as the conditioned (SIF, LIF) H-reflex amplitude over the unconditioned H-reflex amplitude (conditioned/unconditioned), at three PNS intensity conditions (50% Hmax, Hmax, 150% Hmax).

Unconditioned and conditioned (SIF, LIF) H-reflex recruitment curves were plotted at 5 points along the H-reflex recruitment curve (H-threshold, 50% Hmax, Hmax, 150% Hmax, H-endpoint) (Figure 2). To evaluate the magnitude of facilitation along the ascending, peak, and descending portions of the H-reflex recruitment curve, both unconditioned and conditioned (SIF, LIF) H-reflex amplitudes were normalized to each individual’s Mmax amplitude and compared at 3 intensity conditions (50% Hmax, Hmax, 150% Hmax) (Figure 3A).

### Statistical Analyses

Normality was assessed using the Shapiro-Wilk test and quantile-quantile plots. Sphericity was assessed using Mauchly’s test. If sphericity could not be assumed, Greenhouse-Geisser correction was applied to the degrees of freedom. Significant main effects were followed by *post hoc* contrasts and corrected for multiple comparisons using the Bonferroni method. To first confirm the presence of SIF and LIF at a single, standard PNS intensity eliciting an H-reflex equivalent to 20% of Mmax (Nielsen and Petersen, 1995b; Geertsen et al., 2011), a one-way repeated-measures analysis of variance (ANOVA) was used to evaluate the effect of TMS-conditioning on H-reflex amplitude (unconditioned, SIF, LIF). A two-way repeated measures ANOVA was then performed to evaluate the effect of PNS intensity (3 standardized points along the recruitment curve) and TMS-conditioning (unconditioned, SIF, LIF) on the H-reflex amplitudes normalized to Mmax. For the two-way repeated measures ANOVA, we compared 3 intensities along the recruitment curve that represented the ascending limb (50% Hmax), peak (Hmax), and descending limb (150% Hmax). Simple effects analyses were performed to compare the H-reflex amplitudes normalized to Mmax at each of the 3 intensity conditions. For each TMS-conditioning level, pairwise comparisons were performed to compare the magnitude of facilitation between the 3 intensity conditions. A repeated measures two-way ANOVA was performed to assess the effect of PNS intensity and facilitation condition on the facilitation ratio (conditioned/unconditioned H-reflex amplitudes). Additionally, two separate one-way repeated-measures ANOVAs were performed to evaluate the effects of TMS-conditioning (unconditioned, SIF, LIF) on the Hmax/Mmax ratio and the intensity at which Hmax occurred. All statistical tests were run in Statistical Package for the Social Sciences (IBM SPSS version 26) and the critical alpha level was set to *p* < 0.05.

## Results

Complete datasets were collected for 10 of the 11 participants; data from one participant were not included in the analyses due to methodological issues during the experimental session.

### Demonstration of Short- and Long-Interval Facilitation of H-Reflexes at a Single PNS Intensity (20% Mmax)

Confirming short- and long-interval facilitation of the H-reflex at the 20% of Mmax intensity, the one-way repeated measures ANOVA revealed a significant main effect of conditioning (unconditioned, SIF, LIF) on H-reflex amplitude [*F*_(2, 1)_ = 15.51, *p* < 0.001; η^2^*_*p*_* = 0.63]. *Post hoc* pairwise comparisons showed significantly greater H-reflex amplitudes for LIF compared to unconditioned (*p* = 0.002) and SIF compared to unconditioned (*p* = 0.018) at the 20% of Mmax intensity (Figure 1C).

### Evaluation of Short- and Long-Interval Facilitation of H-Reflexes at a Range of PNS Intensities

Across the range of PNS intensities evaluated, both the conditioned H-reflex curves (SIF and LIF conditions) demonstrated larger H-reflex amplitudes compared to the unconditioned H-reflex recruitment curve (Figure 2). The two-way repeated measures ANOVA revealed a significant main effect for facilitation [*F*_(2, 18)_ = 13.50, *p* < 0.001; η^2^*_*p*_* = 0.60], intensity [*F*_(2, 18)_ = 51.34, *p* < 0.001; η^2^*_*p*_* = 0.85], and an interaction between facilitation and intensity [*F*_(4, 36)_ = 4.39, *p* = 0.005; η^2^*_*p*_* = 0.33]. Simple effects analysis revealed a significant effect of TMS-conditioning at the 50% Hmax [*F*_(2, 81)_ = 4.59, *p* = 0.013] and Hmax [*F*_(2, 81)_ = 4.01, *p* = 0.022] intensities. *Post hoc* pairwise comparisons revealed significantly higher H-reflex amplitudes normalized to Mmax for LIF compared to unconditioned at 50% Hmax (*p* = 0.010) and Hmax (*p* = 0.021) (Figure 3A). Additionally, *post hoc* pairwise comparisons revealed significantly greater magnitude of SIF at 50% Hmax compared to 150% Hmax (*p* = 0.005), and significantly greater magnitude of LIF at Hmax compared to 50% Hmax (*p* = 0.025) and 150% Hmax (*p* < 0.001). No other comparisons were significant (*p* > 0.076). To assess the effect of PNS intensity and facilitation condition on facilitation ratios, the two-way repeated measures ANOVA revealed a significant main effect for PNS intensity [*F*_(1.21, 10.91)_ = 6.80, *p* = 0.021; η^2^*_*p*_* = 0.43] and across PNS intensities, there was greater facilitation for the LIF condition compared to the SIF condition [*F*_(1, 9)_ = 5.98, *p* = 0.037; η^2^*_*p*_* = 0.40] (Figure 3B). No significant interaction effect was observed (*p* = 0.086).

### Comparison of Unconditioned and Conditioned Hmax and Intensity Required to Elicit Hmax

**FIGURE 4:**
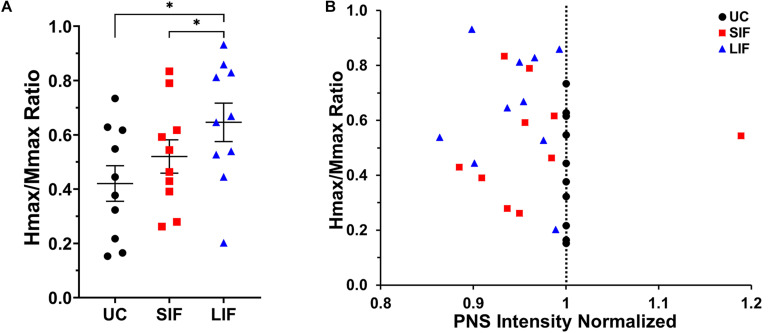
**(A)** The graph above shows the mean and standard error for Hmax values normalized to Mmax for unconditioned (UC), short-interval facilitation (SIF), and long-interval facilitation (LIF). For LIF, the Hmax/Mmax ratio (0.630 ± 0.070) was significantly greater than the Hmax/Mmax ratios for UC (0.429 ± 0.068; *p* = 0.005) and SIF (0.492 ± 0.061; *p* = 0.041). **(B)** Normalized Hmax values as a function of stimulus intensity (normalized to unconditioned). TMS-conditioned Hmax was achieved at a lower stimulus intensity (normalized PNS intensity < 1) compared to unconditioned Hmax for all but one participant.

The one-way repeated measures ANOVA revealed a significant main effect of TMS-conditioning (unconditioned, SIF, LIF) on the Hmax/Mmax ratio [*F*_(2, 18)_ = 10.13, *p* = 0.001; η^2^_*p*_ = 0.53]. *Post hoc* pairwise comparisons showed significantly greater Hmax/Mmax ratio for LIF when compared to unconditioned (*p* = 0.005) and SIF (*p* = 0.041) (Figure 4A). One-way repeated measures ANOVA revealed a significant main effect of conditioning [*F*_(2, 18)_ = 3.554, *p* = 0.050; η^2^*_*p*_* = 0.28] on the intensity used to obtain Hmax, which was normalized to the intensity used to obtain unconditioned Hmax. *Post hoc* pairwise comparisons showed a significantly lower intensity at which Hmax was obtained for LIF compared to unconditioned (*p* = 0.006), suggesting a leftward shift of the H-reflex curve. No significant differences in intensity to elicit Hmax were observed between SIF compared to unconditioned (*p* = 0.815) or SIF compared to LIF (*p* = 0.818). Although we did not find a statistically significant difference in intensity eliciting Hmax between SIF compared to unconditioned, individual participant data revealed that for all but one participant, Hmax occurred at a lower stimulus intensity during the SIF versus the unconditioned condition (Figure 4B). Additionally, all participants demonstrated that Hmax occurred at a lower intensity during LIF versus the unconditioned condition (Figure 4B).

## Discussion

Our results revealed that across participants, at the same PNS intensity used for each condition (UC, SIF, LIF), TMS-conditioned H-reflexes, specifically for LIF, were significantly greater than unconditioned H-reflexes at multiple PNS intensities across the H-reflex recruitment curve. For LIF, proposed to be mediated by indirect, slower descending projections, TMS-conditioned H-reflex amplitudes were significantly larger than unconditioned H-reflex amplitudes on the ascending limb (50% Hmax) and peak (Hmax) of the H-reflex recruitment curve. Additionally, for LIF, we observed a lower PNS intensity needed to elicit the same soleus H-reflex amplitude as UC (i.e., the leftward shift of the conditioned H-reflex recruitment curve). Taken together, our finding that TMS-induced facilitation of soleus H-reflexes occurs at multiple PNS intensities across the H-reflex recruitment curve provides novel insight into the ability of convergent synaptic inputs (from descending cortical and spinal reflex pathways) to augment the physiologic recruitment order of the soleus H-reflex.

Previous studies evaluating SIF and LIF have typically measured H-reflex facilitation at a single PNS intensity, usually an intensity along the ascending limb of the H-reflex curve (e.g., an intensity eliciting an H-reflex amplitude at about 20% of Mmax) (Nielsen et al., 1993; Petersen et al., 2003; Geertsen et al., 2011). Mechanisms that may explain this facilitatory phenomenon in soleus include a decrease in the amount of presynaptic inhibition acting on Ia afferents (Costa et al., 2011), as well as temporal summation of excitatory postsynaptic potentials (EPSPs) from corticospinal projections and/or indirectly from polysynaptic pathways (Nielsen and Petersen, 1995b). To our knowledge, SIF and LIF have not been previously evaluated at a range of PNS intensities. Therefore, the current study provides a novel, systematic, and comprehensive approach that probes convergent sensorimotor inputs on reflex gain within spinal motor circuitry.

During recording of the unconditioned H-reflex curve, trans-synaptic recruitment of LMNs occurs in accordance with the size principle (Pierrot-Deseilligny and Mazevet, 2000; Binboga and Turker, 2012), with smaller diameter LMNs contributing to the compound H-reflex amplitude at lower PNS intensities (at H-threshold and ascending limb of the H-curve), and larger diameter LMNs being recruited at higher PNS intensities (peak of H-curve and descending limb) (Davies et al., 1993; Gorassini et al., 2002). The Hmax/Mmax ratio represents the largest proportion of LMNs that are trans-synaptically recruited in response to the Ia afferent volleys. However, the direct stimulation of motor axons by PNS also simultaneously elicits an antidromic volley that recruits the larger-diameter motor axons before the smaller-diameter motor axons (Gottlieb and Agarwal, 1976; Burke et al., 1993; Funase et al., 1994). The “collision” of these antidromic volleys in the LMNs with the descending orthodromic LMN volley contributes to the reduced amplitude of the H-reflex (e.g., the descending limb of the curve), and ultimately abolishes the H-reflex with only M-waves visible (H-reflex curve endpoint). Based on our findings, during TMS-conditioning, we posit that the TMS-induced descending volleys result in membrane depolarization of the LMNs trans-synaptically, which also follows physiological recruitment order (Bawa and Lemon, 1993). The TMS pulse, although delivered at a sub-threshold intensity, may cause depolarization of a sub-population of LMN cell membranes (Bawa and Lemon, 1993), potentially inducing a greater change in membrane potential of smaller and medium diameter LMN fibers (Davies et al., 1993; Gorassini et al., 2002). Thus, at PNS intensities matched to the *unconditioned, no-TMS* condition, in the presence of TMS-facilitation, the upward shift of the conditioned H-reflex recruitment curves may represent a greater relative contribution of the smaller and medium-diameter LMNs to the compound H-reflex amplitude. We propose that the modulation of recruitment profiles of LMNs contributing to the compound H-reflex explain the upward shift of the conditioned H-reflex recruitment curves (e.g., lower stimulus intensities eliciting increased H-reflex amplitudes across the TMS-conditioned curves).

Another potential explanation for the observed finding is that TMS-conditioning may alter the membrane excitability of the LMN pool effectively lowering the activation threshold such that lower PNS stimulus intensities would recruit a greater proportion of the LMN pool when “primed” by TMS, potentially explaining the leftward shift of the curve. TMS-conditioning may also enable a greater proportion of medium and smaller diameter LMNs to be recruited in the compound H-reflex response, allowing for the measurement of a greater H-reflex amplitude, before occlusion of the H-reflex in the descending limb of the curve. Medium diameter LMNs that may have been “canceled out” by the collision with the antidromic signal for the *unconditioned* condition then could potentially contribute to the compound H-reflex in the SIF and LIF condition, explaining the increase in Hmax with LIF.

SIF of the H-reflex is thought to index the most direct, fastest descending volleys that arrive at the spinal segment prior to the arrival of the Ia afferent signal (Nielsen et al., 1993; Petersen et al., 1998; Taube et al., 2017). The descending cortical pathways that contribute to these observations of “early facilitation” have been described as the fastest, presumably monosynaptic, corticomotoneuronal connections between M1 and the LMN pool (Nielsen and Petersen, 1995b; Butler et al., 2007; Geertsen et al., 2011). Our finding of significant SIF of soleus H-reflexes at the H-reflex amplitude equivalent to 20% Mmax suggests that in contrast to LIF, measuring SIF across multiple PNS intensities may not be necessary to adequately characterize the influence of these direct descending projections on spinal excitability.

In contrast to SIF, LIF is thought to be mediated by indirect, slower descending volleys arriving prior to the Ia afferent signal (Geertsen et al., 2011; Gray et al., 2017). We observed significant H-reflex facilitation for LIF at multiple points along the curve (i.e., at 20% Mmax, 50% Hmax, and Hmax). Previous studies have reported that LIF pathways induce a larger magnitude of facilitation compared to the SIF pathway (Nielsen et al., 1993; Nielsen and Petersen, 1995b). For LIF pathways, greater magnitude of TMS-induced facilitation could be due to contributions from multiple descending pathways that synapse within subcortical, brain stem, and spinal cord regions (Geertsen et al., 2011; Leukel et al., 2015; Niemann et al., 2018). Potentially, the longer latency between TMS and PNS for the LIF condition allows sufficient time for descending volleys of multiple indirect, polysynaptic descending projections to arrive at the spinal segmental level, enabling a greater modulation of membrane excitability of the spinal motoneuron pool.

Previous studies have also suggested that several different corticofugal pathways, such as cortico-rubrospinal or cortico-reticulospinal, may play a role in the facilitation observed at the LIF latency of 10 ms (Nielsen and Petersen, 1995b; Gray et al., 2017; Niemann et al., 2018). A larger magnitude of LIF may also be explained by the arrival of TMS-induced descending volleys at the spinal segmental level at varying times, providing greater opportunity for varied temporal summation of excitatory post-synaptic potentials (EPSPs) (Geertsen et al., 2011; Gray et al., 2017). This could explain why LIF was greater, and occurred at more sites along the H-reflex curve, than SIF. Although it is known that these indirect, polysynaptic pathways are involved in multiple neurophysiological processes (Deliagina et al., 2008; Honeycutt et al., 2013; Fregosi et al., 2017), future studies are required to characterize the unique and combined influences on spinal reflex excitability.

Taken together, our results show that evaluating H-reflex facilitation at multiple PNS intensities reveal additional insights into the mechanisms of descending supraspinal influences on spinal circuitry and reflex gain that may have practical applications for future investigations including individuals with neurologic impairments, such as stroke or spinal cord injury (SCI). Descending corticomotor projections are important components of neural circuitry controlling voluntary movement (Burke et al., 1993; Butler et al., 2007; Sidhu et al., 2012). Current non-invasive neurophysiologic measures, such as standalone single-pulse TMS or H-reflex amplitudes, are unable to specifically evaluate the connections between cortical and spinal circuits in humans, leading to a gap in our understanding of the salient mechanisms underlying recovery of movement associated with rehabilitative interventions. Additionally, evaluating both SIF and LIF could determine whether rehabilitation interventions differentially modulate the excitability of direct and indirect descending projections, an area of training-induced neuroplasticity that is not well-understood. Future studies are needed to examine the influence of other methodological parameters (i.e., muscle activation, posture, task, TMS intensity), and in the context of neurologic conditions (i.e., stroke, SCI), on TMS-induced H-reflex facilitation. Taken together, SIF and LIF may provide valuable neurophysiologic outcome variables for studying rehabilitation-induced neuroplasticity of direct and indirect descending corticomotor projections onto spinal LMNs.

### Limitations

In the current study, the same PNS intensities were used for the unconditioned and conditioned (SIF and LIF) H-reflex recruitment curves for each participant. The same subthreshold TMS intensity, determined at the beginning of the experimental session, was used for the conditioned (SIF and LIF) H-reflex recruitment curves. Furthermore, the EMG sensor placement location, PNS stimulation site, TMS coil location, and muscle activation state were the same for each individual participant across all 3 conditions. Thus, our experimental design was such that H-reflex facilitation observed in the SIF and LIF conditions compared to unconditioned cannot be ascribed to differences in PNS intensity, TMS intensity, or methodological concerns.

However, the current study has limitations. In the current experimental protocol, we interleaved UC, SIF, and LIF and delivered them sequentially and repeatedly over a range of PNS intensities. If LIF always occurring after SIF causes larger magnitude of facilitation, the fact that each LIF was followed by UC and SIF should have minimized a potential order effect. Nevertheless, our results could be influenced by this ordering effect, which can be addressed in future studies by potentially randomizing the delivery order of PNS intensity or facilitation condition. TMS-conditioning of the H-reflex can be used to evaluate the influences of descending corticomotor pathways on spinal reflex excitability, but this technique is limited to muscles in which it is possible to elicit a stable H-reflex. The study is limited by a relatively small sample size although the size is in line with previous studies (Nielsen et al., 1993; Guzman-Lopez et al., 2015; Gray et al., 2017) and our preliminary findings yielded significant results. Findings from the current study provide a proof of concept that will aid in the design and powering of future studies on the influence of convergent sensorimotor inputs within the spinal cord.

We did not objectively assess pain perception during the experiment. Previous findings have indicated that some participants may perceive pain in response to a standardized protocol to elicit soleus H-reflexes (Motl et al., 2002, 2004) but participants in the current study did not report significant discomfort with study procedures. Additionally, in the present study, data were collected while participants were in an active state (maintaining a sustained low-level volitional muscle activation) and in a specific body position (seated). Throughout the experiment, we monitored and displayed to the participant real-time visual feedback regarding the ongoing background EMG activation with respect to the target EMG. However, small variations in background EMG may have influence the study results. Previous studies have also shown that muscle activation state, body position and length, and posture can influence both cortical and spinal excitability (Nielsen et al., 1993; Nielsen and Petersen, 1995a; Guzman-Lopez et al., 2015; van den Bos et al., 2017).

Here, we assessed SIF and LIF at a single standardized ISI, respectively, for all participants that does not account for inter-individual variability in conduction velocities, limb length, or other individual characteristics. Individualizing the ISI for each participant to optimize the magnitude of facilitation (Nielsen et al., 1993; Taube et al., 2017), and accounting for each individuals’ body or limb length parameters, as well as nerve conduction velocities, would be valuable directions for future study. Finally, during collection of unconditioned and TMS-conditioned H-reflex recruitment curves, H-reflex responses were recorded once at each increasing PNS intensity for all conditions (UC, SIF, LIF) due to experimental time limitations. Thus, evaluating SIF and LIF using a greater number of trials throughout the H-reflex recruitment curve, as well as collecting a wider range of PNS intensities across the H-reflex recruitment curve (i.e., prior to H-reflex onset), could establish reliability and reproducibility, and may be important methodological considerations for future studies.

## Conclusion

The current study findings demonstrated that TMS-conditioning of soleus H-reflexes resulted in greater magnitude of facilitation (LIF) at multiple PNS intensities on the H-reflex recruitment curve (50% Hmax, Hmax), and that TMS-conditioning resulted in an increased Hmax/Mmax ratio in the LIF condition, inducing a leftward shift of the H-reflex recruitment curve. Our findings suggest that evaluating SIF and LIF over a range of PNS intensities offers a non-invasive approach to characterize descending corticomotor influences on spinal circuit activity. Additionally, our findings further elucidate the ability of convergent sensorimotor inputs to modulate the recruitment profiles of spinal LMNs. Further optimization of approaches used to characterize integration of ascending sensory and descending motor signals at the spinal segmental level offer novel opportunities to improve our understanding of neurophysiologic mechanisms of abnormal spinal circuit activity and motor function.

## Data Availability Statement

The original contributions presented in the study are included in the article/supplementary material, further inquiries can be directed to the corresponding author.

## Ethics Statement

The studies involving human participants were reviewed and approved by the Emory Institutional Review Board (IRB)—IRB00067708; Rebecca Rousselle (rebecca.rousselle@emory.edu). The patients/participants provided their written informed consent to participate in this study.

## Author Contributions

CM, TK, and MB conceived and designed research. CM, MH, and TK performed the experiments. AL, MH, and CM analyzed the data, prepared the figures, and drafted the manuscript. AL, MH, TK, MB, and CM interpreted results of experiments. AL, JX, MH, TK, and MB edited and revised the manuscript and approved final version of manuscript.

## Conflict of Interest

The authors declare that the research was conducted in the absence of any commercial or financial relationships that could be construed as a potential conflict of interest.

## References

[B1] Bagust J. Kelly M. E. Kerkut G. A. (1985). An isolated mammalian brainstem-spinal cord preparation suitable for the investigation of descending control of motor activity. *Brain Res.* 327 370–374. 10.1016/0006-8993(85)91539-2 3986517

[B2] Bakheit A. M. Maynard V. Shaw S. (2005). The effects of isotonic and isokinetic muscle stretch on the excitability of the spinal alpha motor neurones in patients with muscle spasticity. *Eur. J. Neurol.* 12 719–724. 10.1111/j.1468-1331.2005.01068.x 16128875

[B3] Barker A. T. Jalinous R. Freeston I. L. (1985). Non-invasive magnetic stimulation of human motor cortex. *Lancet* 1 1106–1107. 10.1016/s0140-6736(85)92413-4 2860322

[B4] Bawa P. Lemon R. N. (1993). Recruitment of motor units in response to transcranial magnetic stimulation in man. *J. Physiol.* 471 445–464. 10.1113/jphysiol.1993.sp019909 8120816 PMC1143970

[B5] Bhagchandani N. Schindler-Ivens S. (2012). Reciprocal inhibition post-stroke is related to reflex excitability and movement ability. *Clin. Neurophysiol.* 123 2239–2246. 10.1016/j.clinph.2012.04.023 22613030 PMC3592335

[B6] Binboga E. Turker K. S. (2012). Compound group I excitatory input is differentially distributed to human soleus motoneurons. *Clin. Neurophysiol.* 123 2192–2199. 10.1016/j.clinph.2012.04.005 22608971

[B7] Burke D. (2016). Clinical uses of H reflexes of upper and lower limb muscles. *Clin. Neurophysiol. Pract.* 1 9–17. 10.1016/j.cnp.2016.02.003 30214954 PMC6123946

[B8] Burke D. Hicks R. Gandevia S. C. Stephen J. Woodforth I. Crawford M. (1993). Direct comparison of corticospinal volleys in human subjects to transcranial magnetic and electrical stimulation. *J. Physiol.* 470 383–393. 10.1113/jphysiol.1993.sp019864 8068071 PMC1143923

[B9] Buschges A. (2017). Controlling the ‘simple’ - descending signals from the brainstem command the sign of a stretch reflex in a vertebrate spinal cord. *J. Physiol.* 595 625–626. 10.1113/jp273352 28145003 PMC5285624

[B10] Butler J. E. Larsen T. S. Gandevia S. C. Petersen N. T. (2007). The nature of corticospinal paths driving human motoneurones during voluntary contractions. *J. Physiol.* 584 651–659. 10.1113/jphysiol.2007.134205 17702821 PMC2277157

[B11] Cecen S. Niazi I. K. Nedergaard R. W. Cade A. Allen K. Holt K. (2018). Posture modulates the sensitivity of the H-reflex. *Exp. Brain Res.* 236 829–835. 10.1007/s00221-018-5182-x 29349480

[B12] Cerqueira V. De Mendonca A. Minez A. Dias A. R. De Carvalho M. (2006). Does caffeine modify corticomotor excitability? *Neurophysiol. Clin.* 36 219–226. 10.1016/j.neucli.2006.08.005 17095411

[B13] Chen H. H. Hippenmeyer S. Arber S. Frank E. (2003). Development of the monosynaptic stretch reflex circuit. *Curr. Opin. Neurobiol.* 13 96–102. 10.1016/s0959-4388(03)00006-0 12593987

[B14] Costa J. Guzman J. Valldeoriola F. Rumia J. Tolosa E. Casanova-Molla J. (2011). Modulation of the soleus H reflex by electrical subcortical stimuli in humans. *Exp. Brain Res.* 212 439–448. 10.1007/s00221-011-2750-8 21656215

[B15] Crone C. Hultborn H. Mazieres L. Morin C. Nielsen J. Pierrot-Deseilligny E. (1990). Sensitivity of monosynaptic test reflexes to facilitation and inhibition as a function of the test reflex size: a study in man and the cat. *Exp. Brain Res.* 81 35–45. 2394229 10.1007/BF00230098

[B16] Davies L. Wiegner A. W. Young R. R. (1993). Variation in firing order of human soleus motoneurons during voluntary and reflex activation. *Brain Res.* 602 104–110. 10.1016/0006-8993(93)90248-l 8448646

[B17] Deliagina T. G. Beloozerova I. N. Zelenin P. V. Orlovsky G. N. (2008). Spinal and supraspinal postural networks. *Brain Res. Rev.* 57 212–221. 10.1016/j.brainresrev.2007.06.017 17822773 PMC2204048

[B18] Di Lazzaro V. Rothwell J. C. (2014). Corticospinal activity evoked and modulated by non-invasive stimulation of the intact human motor cortex. *J. Physiol.* 592 4115–4128. 10.1113/jphysiol.2014.274316 25172954 PMC4215763

[B19] Fregosi M. Contestabile A. Hamadjida A. Rouiller E. M. (2017). Corticobulbar projections from distinct motor cortical areas to the reticular formation in macaque monkeys. *Eur. J. Neurosci.* 45 1379–1395. 10.1111/ejn.13576 28394483

[B20] Funase K. Imanaka K. Nishihira Y. (1994). Excitability of the soleus motoneuron pool revealed by the developmental slope of the H-reflex as reflex gain. *Electromyogr. Clin. Neurophysiol.* 34 477–489. 7882891

[B21] Gassel M. M. (1969). Monosynaptic reflexes (H-reflex) and motoneurone excitability in man. *Dev. Med. Child. Neurol.* 11 193–197. 10.1111/j.1469-8749.1969.tb01417.x 5787717

[B22] Gassel M. M. Diamantopoulos E. (1966). Mechanically and electrically elicited monosynaptic reflexes in man. *J. Appl. Physiol.* 21 1053–1058. 10.1152/jappl.1966.21.3.1053 5912723

[B23] Geertsen S. S. Van De Ruit M. Grey M. J. Nielsen J. B. (2011). Spinal inhibition of descending command to soleus motoneurons is removed prior to dorsiflexion. *J. Physiol.* 589 5819–5831. 10.1113/jphysiol.2011.214387 21986208 PMC3249052

[B24] Geertsen S. S. Zuur A. T. Nielsen J. B. (2010). Voluntary activation of ankle muscles is accompanied by subcortical facilitation of their antagonists. *J. Physiol.* 588 2391–2402. 10.1113/jphysiol.2010.190678 20457734 PMC2915515

[B25] Gorassini M. Yang J. F. Siu M. Bennett D. J. (2002). Intrinsic activation of human motoneurons: possible contribution to motor unit excitation. *J. Neurophysiol.* 87 1850–1858. 10.1152/jn.00024.2001 11929906

[B26] Gottlieb G. L. Agarwal G. C. (1976). Extinction of the Hoffmann reflex by antidromic conduction. *Electroencephalogr. Clin. Neurophysiol.* 41 19–24. 10.1016/0013-4694(76)90211-x 58765

[B27] Granit R. Job C. (1952). Electromyographic and monosynaptic definition of reflex excitability during muscle stretch. *J. Neurophysiol.* 15 409–420. 10.1152/jn.1952.15.5.409 12991106

[B28] Gray W. A. Sabatier M. J. Kesar T. M. Borich M. R. (2017). Establishing between-session reliability of TMS-conditioned soleus H-reflexes. *Neurosci. Lett.* 640 47–52. 10.1016/j.neulet.2017.01.032 28093306 PMC5315025

[B29] Groppa S. Oliviero A. Eisen A. Quartarone A. Cohen L. G. Mall V. (2012). A practical guide to diagnostic transcranial magnetic stimulation: report of an IFCN committee. *Clin. Neurophysiol.* 123 858–882. 22349304 10.1016/j.clinph.2012.01.010 PMC4890546

[B30] Guzman-Lopez J. Selvi A. Sola-Valls N. Casanova-Molla J. Valls-Sole J. (2015). Effects of postural and voluntary muscle contraction on modulation of the soleus H reflex by transcranial magnetic stimulation. *Exp. Brain Res.* 233 3425–3431. 10.1007/s00221-015-4417-3 26289484

[B31] Hallett M. (2007). Transcranial magnetic stimulation: a primer. *Neuron* 55 187–199. 10.1016/j.neuron.2007.06.026 17640522

[B32] Honeycutt C. F. Kharouta M. Perreault E. J. (2013). Evidence for reticulospinal contributions to coordinated finger movements in humans. *J. Neurophysiol.* 110 1476–1483. 10.1152/jn.00866.2012 23825395 PMC4042417

[B33] Kesar T. M. Eicholtz S. Lin B. J. Wolf S. L. Borich M. R. (2018). Effects of posture and coactivation on corticomotor excitability of ankle muscles. *Restor. Neurol. Neurosci.* 36 131–146. 10.3233/rnn-170773 29439363 PMC5901671

[B34] Knikou M. (2008). The H-reflex as a probe: pathways and pitfalls. *J. Neurosci. Methods* 171 1–12. 10.1016/j.jneumeth.2008.02.012 18394711

[B35] Komiyama T. Kawai K. Fumoto M. (1999). The excitability of a motoneuron pool assessed by the H-reflex method is correlated with the susceptibility of Ia terminals to repetitive discharges in humans. *Brain Res.* 826 317–320. 10.1016/s0006-8993(99)01301-3 10224313

[B36] Leukel C. Taube W. Rittweger J. Gollhofer A. Ducos M. Weber T. (2015). Changes in corticospinal transmission following 8weeks of ankle joint immobilization. *Clin. Neurophysiol.* 126 131–139. 10.1016/j.clinph.2014.04.002 24794515

[B37] Mazzocchio R. Scarfo G. B. Mariottini A. Muzii V. F. Palma L. (2001). Recruitment curve of the soleus H-reflex in chronic back pain and lumbosacral radiculopathy. *BMC Musculoskelet Disord.* 2:4. 10.1186/1471-2474-2-4 PMC60003 11722799

[B38] Meunier S. Pierrot-Deseilligny E. (1989). Gating of the afferent volley of the monosynaptic stretch reflex during movement in man. *J. Physiol.* 419 753–763. 10.1113/jphysiol.1989.sp017896 2621649 PMC1190031

[B39] Meunier S. Pierrot-Deseilligny E. (1998). Cortical control of presynaptic inhibition of Ia afferents in humans. *Exp. Brain Res.* 119 415–426. 10.1007/s002210050357 9588776

[B40] Milanov I. G. (2000). Evaluation of the presynaptic inhibition by comparing the amplitudes of H reflexes and F waves. Is it possible? *Electromyogr. Clin. Neurophysiol.* 40 491–495. 11155542

[B41] Motl R. W. O’connor P. J. Boyd C. M. Dishman R. K. (2002). Low intensity pain reported during elicitation of the H-reflex: no effects of trait anxiety and high intensity cycling exercise. *Brain Res.* 951 53–58. 10.1016/s0006-8993(02)03134-7 12231456

[B42] Motl R. W. O’connor P. J. Knowles B. D. (2004). No effect of skin anesthesia on pain intensity ratings associated with elicitation of the H-reflex in the soleus muscle. *Int. J. Neurosci.* 114 1549–1560. 10.1080/00207450490509276 15512838

[B43] Nielsen J. Petersen N. (1995a). Changes in the effect of magnetic brain stimulation accompanying voluntary dynamic contraction in man. *J. Physiol.* 484(Pt 3) 777–789. 10.1113/jphysiol.1995.sp020703 7623292 PMC1157960

[B44] Nielsen J. Petersen N. (1995b). Evidence favouring different descending pathways to soleus motoneurones activated by magnetic brain stimulation in man. *J. Physiol.* 486(Pt 3) 779–788. 10.1113/jphysiol.1995.sp020853 7473238 PMC1156565

[B45] Nielsen J. Petersen N. Deuschl G. Ballegaard M. (1993). Task-related changes in the effect of magnetic brain stimulation on spinal neurones in man. *J. Physiol.* 471 223–243. 10.1113/jphysiol.1993.sp019899 8120805 PMC1143960

[B46] Niemann N. Wiegel P. Kurz A. Rothwell J. C. Leukel C. (2018). Assessing TMS-induced D and I waves with spinal H-reflexes. *J. Neurophysiol.* 119 933–943. 10.1152/jn.00671.2017 29142099

[B47] Perez M. A. Cohen L. G. (2009). The corticospinal system and transcranial magnetic stimulation in stroke. *Top. Stroke Rehabil.* 16 254–269. 10.1310/tsr1604-254 19740731 PMC4886556

[B48] Petersen N. Christensen L. O. Nielsen J. (1998). The effect of transcranial magnetic stimulation on the soleus H reflex during human walking. *J. Physiol.* 513(Pt 2) 599–610. 10.1111/j.1469-7793.1998.599bb.x 9807007 PMC2231281

[B49] Petersen N. T. Pyndt H. S. Nielsen J. B. (2003). Investigating human motor control by transcranial magnetic stimulation. *Exp. Brain Res.* 152 1–16. 10.1007/s00221-003-1537-y 12879177

[B50] Pierrot-Deseilligny E. Mazevet D. (2000). The monosynaptic reflex: a tool to investigate motor control in humans. Interest and limits. *Neurophysiol. Clin.* 30 67–80. 10.1016/s0987-7053(00)00062-9 10812576

[B51] Pyndt H. S. Nielsen J. B. (2003). Modulation of transmission in the corticospinal and group Ia afferent pathways to soleus motoneurons during bicycling. *J. Neurophysiol.* 89 304–314. 10.1152/jn.00386.2002 12522181

[B52] Ross H. G. Cleveland S. Haase J. (1972). Quantitative relation of Renshaw cell discharges to monosynaptic reflex height. *Pflugers Arch.* 332 73–79. 10.1007/bf00603815 5063043

[B53] Ross H. G. Cleveland S. Haase J. (1975). Contribution of single motoneurons to renshaw cell activity. *Neurosci. Lett.* 1 105–108. 10.1016/0304-3940(75)90053-1 19604761

[B54] Ross H. G. Cleveland S. Haase J. (1976). Quantitative relation between discharge frequencies of a Renshaw cell and an intracellularly depolarized motoneuron. *Neurosci. Lett.* 3 129–132. 10.1016/0304-3940(76)90081-1 19604874

[B55] Rossini P. M. Burke D. Chen R. Cohen L. G. Daskalakis Z. Di Iorio R. (2015). Non-invasive electrical and magnetic stimulation of the brain, spinal cord, roots and peripheral nerves: Basic principles and procedures for routine clinical and research application. An updated report from an I.F.C.N. Committee. *Clin. Neurophysiol.* 126 1071–1107. 10.1016/j.clinph.2015.02.001 25797650 PMC6350257

[B56] Schieppati M. (1987). The Hoffmann reflex: a means of assessing spinal reflex excitability and its descending control in man. *Prog. Neurobiol.* 28 345–376. 10.1016/0301-0082(87)90007-4 3588965

[B57] Schindler-Ivens S. Brown D. A. Lewis G. N. Nielsen J. B. Ondishko K. L. Wieser J. (2008). Soleus H-reflex excitability during pedaling post-stroke. *Exp. Brain Res.* 188 465–474. 10.1007/s00221-008-1373-1 18427793

[B58] Sidhu S. K. Hoffman B. W. Cresswell A. G. Carroll T. J. (2012). Corticospinal contributions to lower limb muscle activity during cycling in humans. *J. Neurophysiol.* 107 306–314. 10.1152/jn.00212.2011 22013236

[B59] Taube W. Leukel C. Nielsen J. B. Lundbye-Jensen J. (2017). Non-invasive assessment of changes in corticomotoneuronal transmission in humans. *J. Vis. Exp.* 52663. 10.3791/52663 28570549 PMC5608135

[B60] van den Bos M. A. Geevasinga N. Menon P. Burke D. Kiernan M. C. Vucic S. (2017). Physiological processes influencing motor-evoked potential duration with voluntary contraction. *J. Neurophysiol.* 117 1156–1162. 10.1152/jn.00832.2016 28031404 PMC5340882

[B61] Walsh D. M. Lowe A. S. Mccormack K. Willer J. C. Baxter G. D. Allen J. M. (1998). Transcutaneous electrical nerve stimulation: effect on peripheral nerve conduction, mechanical pain threshold, and tactile threshold in humans. *Arch. Phys. Med. Rehabil.* 79 1051–1058. 10.1016/s0003-9993(98)90170-8 9749683

[B62] Walton C. Kalmar J. Cafarelli E. (2003). Caffeine increases spinal excitability in humans. *Muscle Nerve* 28 359–364. 10.1002/mus.10457 12929197

[B63] Wang Z. Li L. Frank E. (2012). The role of muscle spindles in the development of the monosynaptic stretch reflex. *J. Neurophysiol.* 108 83–90. 10.1152/jn.00074.2012 22490553 PMC3434619

